# Supplementary material for: Potential changes in the extent of suitable habitats for geladas (Theropithecus gelada) in the Anthropocene
Source: BMC Ecol Evol. 2023 Nov 3;23:65. doi: 10.1186/s12862-023-02173-3 (PMC10623689; doi:10.1186/s12862-023-02173-3)
Supplement: Supplementary file 1 — Supplementary Material 1 [file 12862_2023_2173_MOESM1_ESM.docx]

**Supplementary material**

**Potential changes in the extent of suitable habitats for geladas (Theropithecus gelada) in the Anthropocene**

Ahmed Seid Ahmed, Desalegn Chala, Chala Adugna Kufa, Anagaw Atickem, , Afework Bekele, Jens-Christian Svenning, Dietmar Zinner

One table, four figures

**Table S1** Occurrence locations of gelada (*Theropithecus gelada*)

| # | Region | Taxon | Latitude | Longitude |
| --- | --- | --- | --- | --- |
| 1 | north | *T. g. gelada* | 14.33333 | 39.48333 |
| 2 | north | *T. g. gelada* | 14.16667 | 39.05000 |
| 3 | north | *T. g. gelada* | 14.13333 | 38.71667 |
| 4 | north | *T. g. gelada* | 14.11667 | 39.43333 |
| 5 | north | *T. g. gelada* | 13.87000 | 39.74000 |
| 6 | north | *T. g. gelada* | 13.75000 | 38.53333 |
| 7 | north | *T. g. gelada* | 13.75000 | 38.08333 |
| 8 | north | *T. g. gelada* | 13.71667 | 38.38333 |
| 9 | north | *T. g. gelada* | 13.66667 | 38.41667 |
| 10 | north | *T. g. gelada* | 13.50000 | 38.50000 |
| 11 | north | *T. g. gelada* | 13.49278 | 38.44361 |
| 12 | north | *T. g. gelada* | 13.40000 | 38.20000 |
| 13 | north | *T. g. gelada* | 13.37359 | 38.29285 |
| 14 | north | *T. g. gelada* | 13.35755 | 38.28735 |
| 15 | north | *T. g. gelada* | 13.34038 | 38.09607 |
| 16 | north | *T. g. gelada* | 13.33282 | 38.42881 |
| 17 | north | *T. g. gelada* | 13.30645 | 38.26414 |
| 18 | north | *T. g. gelada* | 13.30402 | 38.42657 |
| 19 | north | *T. g. gelada* | 13.30163 | 38.29574 |
| 20 | north | *T. g. gelada* | 13.30000 | 39.41667 |
| 21 | north | *T. g. gelada* | 13.28391 | 38.12718 |
| 22 | north | *T. g. gelada* | 13.27725 | 38.08100 |
| 23 | north | *T. g. gelada* | 13.27675 | 38.34958 |
| 24 | north | *T. g. gelada* | 13.27604 | 38.09801 |
| 25 | north | *T. g. gelada* | 13.27122 | 38.03430 |
| 26 | north | *T. g. gelada* | 13.27090 | 38.10860 |
| 27 | north | *T. g. gelada* | 13.26603 | 38.14797 |
| 28 | north | *T. g. gelada* | 13.26601 | 38.07775 |
| 29 | north | *T. g. gelada* | 13.26223 | 38.19221 |
| 30 | north | *T. g. gelada* | 13.25511 | 38.20663 |
| 31 | north | *T. g. gelada* | 13.25195 | 38.34669 |
| 32 | north | *T. g. gelada* | 13.25140 | 38.37314 |
| 33 | north | *T. g. gelada* | 13.25000 | 38.25000 |
| 34 | north | *T. g. gelada* | 13.25000 | 38.23333 |
| 35 | north | *T. g. gelada* | 13.25000 | 38.18333 |
| 36 | north | *T. g. gelada* | 13.25000 | 38.15000 |
| 37 | north | *T. g. gelada* | 13.25000 | 38.08333 |
| 38 | north | *T. g. gelada* | 13.25000 | 38.03333 |
| 39 | north | *T. g. gelada* | 13.25000 | 38.00000 |
| 40 | north | *T. g. gelada* | 13.24639 | 37.89528 |
| 41 | north | *T. g. gelada* | 13.24545 | 38.16689 |
| 42 | north | *T. g. gelada* | 13.24078 | 38.36204 |
| 43 | north | *T. g. gelada* | 13.23533 | 38.02050 |
| 44 | north | *T. g. gelada* | 13.23333 | 38.48333 |
| 45 | north | *T. g. gelada* | 13.23333 | 38.41667 |
| 46 | north | *T. g. gelada* | 13.23145 | 38.03955 |
| 47 | north | *T. g. gelada* | 13.23086 | 37.99430 |
| 48 | north | *T. g. gelada* | 13.23063 | 38.06791 |
| 49 | north | *T. g. gelada* | 13.23021 | 38.08360 |
| 50 | north | *T. g. gelada* | 13.22658 | 38.26053 |
| 51 | north | *T. g. gelada* | 13.22439 | 38.02448 |
| 52 | north | *T. g. gelada* | 13.21667 | 38.15000 |
| 53 | north | *T. g. gelada* | 13.21287 | 38.30383 |
| 54 | north | *T. g. gelada* | 13.20899 | 37.99584 |
| 55 | north | *T. g. gelada* | 13.20833 | 38.11667 |
| 56 | north | *T. g. gelada* | 13.20569 | 37.98582 |
| 57 | north | *T. g. gelada* | 13.20300 | 37.88800 |
| 58 | north | *T. g. gelada* | 13.20050 | 38.08333 |
| 59 | north | *T. g. gelada* | 13.19434 | 38.18943 |
| 60 | north | *T. g. gelada* | 13.16667 | 38.00000 |
| 61 | north | *T. g. gelada* | 13.16550 | 38.07260 |
| 62 | north | *T. g. gelada* | 13.15323 | 37.84451 |
| 63 | north | *T. g. gelada* | 13.14319 | 37.84423 |
| 64 | north | *T. g. gelada* | 13.13190 | 37.93228 |
| 65 | north | *T. g. gelada* | 13.12900 | 37.94341 |
| 66 | north | *T. g. gelada* | 13.12636 | 37.83481 |
| 67 | north | *T. g. gelada* | 13.12138 | 37.93323 |
| 68 | north | *T. g. gelada* | 13.11696 | 38.46255 |
| 69 | north | *T. g. gelada* | 13.08200 | 38.43640 |
| 70 | north | *T. g. gelada* | 13.08191 | 38.50800 |
| 71 | north | *T. g. gelada* | 13.06667 | 38.75000 |
| 72 | north | *T. g. gelada* | 13.02274 | 38.10593 |
| 73 | north | *T. g. gelada* | 13.00349 | 38.10598 |
| 74 | north | *T. g. gelada* | 12.98333 | 37.75000 |
| 75 | north | *T. g. gelada* | 12.97711 | 38.35778 |
| 76 | north | *T. g. gelada* | 12.97380 | 38.10574 |
| 77 | north | *T. g. gelada* | 12.83333 | 37.75000 |
| 78 | north | *T. g. gelada* | 12.77207 | 37.55751 |
| 79 | north | *T. g. gelada* | 12.76903 | 37.60704 |
| 80 | north | *T. g. gelada* | 12.70277 | 37.59908 |
| 81 | north | *T. g. gelada* | 12.61667 | 37.45000 |
| 82 | north | *T. g. gelada* | 12.50000 | 37.50000 |
| 83 | central | *T. g. obscurus* | 12.32977 | 38.89366 |
| 84 | central | *T. g. obscurus* | 12.20000 | 37.98333 |
| 85 | central | *T. g. obscurus* | 12.15469 | 39.17117 |
| 86 | central | *T. g. obscurus* | 12.15434 | 39.17168 |
| 87 | central | *T. g. obscurus* | 12.14825 | 39.18215 |
| 88 | central | *T. g. obscurus* | 12.14565 | 39.18215 |
| 89 | central | *T. g. obscurus* | 12.14531 | 39.18227 |
| 90 | central | *T. g. obscurus* | 12.13892 | 39.18552 |
| 91 | central | *T. g. obscurus* | 12.13159 | 39.20464 |
| 92 | central | *T. g. obscurus* | 12.11759 | 39.18675 |
| 93 | central | *T. g. obscurus* | 12.11667 | 39.46667 |
| 94 | central | *T. g. obscurus* | 12.09639 | 39.37812 |
| 95 | central | *T. g. obscurus* | 12.04750 | 39.09624 |
| 96 | central | *T. g. obscurus* | 12.03132 | 38.89378 |
| 97 | central | *T. g. obscurus* | 12.02394 | 39.39683 |
| 98 | central | *T. g. obscurus* | 12.01667 | 39.05000 |
| 99 | central | *T. g. obscurus* | 12.00000 | 39.00000 |
| 100 | central | *T. g. obscurus* | 11.86372 | 39.19617 |
| 101 | central | *T. g. obscurus* | 11.83333 | 38.08333 |
| 102 | central | *T. g. obscurus* | 11.81667 | 38.65786 |
| 103 | central | *T. g. obscurus* | 11.81257 | 38.67056 |
| 104 | central | *T. g. obscurus* | 11.81100 | 38.69572 |
| 105 | central | *T. g. obscurus* | 11.80884 | 38.68606 |
| 106 | central | *T. g. obscurus* | 11.73286 | 38.89390 |
| 107 | central | *T. g. obscurus* | 11.73186 | 38.18903 |
| 108 | central | *T. g. obscurus* | 11.70341 | 38.23743 |
| 109 | central | *T. g. obscurus* | 11.69748 | 39.25106 |
| 110 | central | *T. g. obscurus* | 11.60368 | 38.92768 |
| 111 | central | *T. g. obscurus* | 11.59543 | 38.94091 |
| 112 | central | *T. g. obscurus* | 11.55421 | 39.22692 |
| 113 | central | *T. g. obscurus* | 11.55181 | 39.10517 |
| 114 | central | *T. g. obscurus* | 11.53333 | 39.21667 |
| 115 | central | *T. g. obscurus* | 11.51261 | 39.01186 |
| 116 | central | *T. g. obscurus* | 11.49615 | 38.93973 |
| 117 | central | *T. g. obscurus* | 11.48385 | 38.79960 |
| 118 | central | *T. g. obscurus* | 11.47721 | 38.97112 |
| 119 | central | *T. g. obscurus* | 11.46081 | 38.82572 |
| 120 | central | *T. g. obscurus* | 11.45757 | 38.84944 |
| 121 | central | *T. g. obscurus* | 11.45091 | 39.24459 |
| 122 | central | *T. g. obscurus* | 11.44982 | 38.99535 |
| 123 | central | *T. g. obscurus* | 11.44982 | 38.94951 |
| 124 | central | *T. g. obscurus* | 11.43439 | 38.89401 |
| 125 | central | *T. g. obscurus* | 11.43333 | 39.31667 |
| 126 | central | *T. g. obscurus* | 11.38629 | 39.24910 |
| 127 | central | *T. g. obscurus* | 11.36742 | 39.24329 |
| 128 | central | *T. g. obscurus* | 11.25000 | 39.58333 |
| 129 | central | *T. g. obscurus* | 11.23220 | 39.21514 |
| 130 | central | *T. g. obscurus* | 11.16983 | 39.24077 |
| 131 | central | *T. g. obscurus* | 11.11667 | 39.71667 |
| 132 | central | *T. g. obscurus* | 11.11210 | 39.14120 |
| 133 | central | *T. g. obscurus* | 11.07247 | 39.27562 |
| 134 | central | *T. g. obscurus* | 11.06100 | 39.66312 |
| 135 | central | *T. g. obscurus* | 11.06007 | 39.66022 |
| 136 | central | *T. g. obscurus* | 11.05917 | 39.66114 |
| 137 | central | *T. g. obscurus* | 11.05809 | 39.66176 |
| 138 | central | *T. g. obscurus* | 10.95747 | 38.68090 |
| 139 | central | *T. g. obscurus* | 10.95197 | 38.66504 |
| 140 | central | *T. g. obscurus* | 10.91760 | 38.79465 |
| 141 | central | *T. g. obscurus* | 10.89623 | 38.85149 |
| 142 | central | *T. g. obscurus* | 10.89421 | 38.80419 |
| 143 | central | *T. g. obscurus* | 10.89290 | 38.82110 |
| 144 | central | *T. g. obscurus* | 10.88725 | 37.51831 |
| 145 | central | *T. g. obscurus* | 10.86667 | 38.75000 |
| 146 | central | *T. g. obscurus* | 10.85150 | 38.79275 |
| 147 | central | *T. g. obscurus* | 10.85028 | 38.68679 |
| 148 | central | *T. g. obscurus* | 10.74129 | 39.18177 |
| 149 | central | *T. g. obscurus* | 10.73444 | 38.78564 |
| 150 | central | *T. g. obscurus* | 10.73353 | 38.88564 |
| 151 | central | *T. g. obscurus* | 10.72312 | 39.29682 |
| 152 | central | *T. g. obscurus* | 10.69457 | 39.32644 |
| 153 | central | *T. g. obscurus* | 10.69262 | 39.36281 |
| 154 | central | *T. g. obscurus* | 10.68437 | 39.18207 |
| 155 | central | *T. g. obscurus* | 10.67874 | 39.71903 |
| 156 | central | *T. g. obscurus* | 10.67739 | 39.23229 |
| 157 | central | *T. g. obscurus* | 10.67483 | 39.31803 |
| 158 | central | *T. g. obscurus* | 10.66667 | 37.95000 |
| 159 | central | *T. g. obscurus* | 10.65850 | 39.41592 |
| 160 | central | *T. g. obscurus* | 10.65739 | 39.25292 |
| 161 | central | *T. g. obscurus* | 10.65000 | 37.75000 |
| 162 | central | *T. g. obscurus* | 10.63899 | 39.80591 |
| 163 | central | *T. g. obscurus* | 10.63879 | 39.13340 |
| 164 | central | *T. g. obscurus* | 10.63422 | 39.17714 |
| 165 | central | *T. g. obscurus* | 10.61601 | 39.33640 |
| 166 | central | *T. g. obscurus* | 10.61250 | 39.68081 |
| 167 | central | *T. g. obscurus* | 10.58771 | 39.63434 |
| 168 | central | *T. g. obscurus* | 10.58753 | 39.44739 |
| 169 | central | *T. g. obscurus* | 10.56086 | 39.59470 |
| 170 | central | *T. g. obscurus* | 10.55490 | 39.44455 |
| 171 | central | *T. g. obscurus* | 10.55338 | 39.68351 |
| 172 | central | *T. g. obscurus* | 10.54603 | 39.56152 |
| 173 | central | *T. g. obscurus* | 10.50315 | 39.54473 |
| 174 | central | *T. g. obscurus* | 10.50307 | 39.57781 |
| 175 | central | *T. g. obscurus* | 10.47395 | 39.51856 |
| 176 | central | *T. g. obscurus* | 10.45169 | 39.17690 |
| 177 | central | *T. g. obscurus* | 10.42464 | 39.80154 |
| 178 | central | *T. g. obscurus* | 10.41404 | 39.74936 |
| 179 | central | *T. g. obscurus* | 10.41124 | 39.77116 |
| 180 | central | *T. g. obscurus* | 10.40927 | 39.26444 |
| 181 | central | *T. g. obscurus* | 10.39131 | 39.38805 |
| 182 | central | *T. g. obscurus* | 10.39125 | 39.41584 |
| 183 | central | *T. g. obscurus* | 10.36555 | 39.48019 |
| 184 | central | *T. g. obscurus* | 10.35000 | 39.78333 |
| 185 | central | *T. g. obscurus* | 10.33700 | 39.47710 |
| 186 | central | *T. g. obscurus* | 10.32744 | 39.80490 |
| 187 | central | *T. g. obscurus* | 10.32593 | 39.20831 |
| 188 | central | *T. g. obscurus* | 10.31758 | 39.80493 |
| 189 | central | *T. g. obscurus* | 10.31383 | 39.81494 |
| 190 | central | *T. g. obscurus* | 10.30559 | 39.29125 |
| 191 | central | *T. g. obscurus* | 10.30094 | 39.81064 |
| 192 | central | *T. g. obscurus* | 10.30018 | 39.19937 |
| 193 | central | *T. g. obscurus* | 10.29453 | 39.25761 |
| 194 | central | *T. g. obscurus* | 10.29119 | 39.78599 |
| 195 | central | *T. g. obscurus* | 10.26238 | 39.08645 |
| 196 | central | *T. g. obscurus* | 10.25106 | 39.07492 |
| 197 | central | *T. g. obscurus* | 10.25000 | 40.00000 |
| 198 | central | *T. g. obscurus* | 10.23347 | 39.11314 |
| 199 | central | *T. g. obscurus* | 10.23256 | 39.17198 |
| 200 | central | *T. g. obscurus* | 10.22663 | 39.09321 |
| 201 | central | *T. g. obscurus* | 10.22656 | 39.15349 |
| 202 | central | *T. g. obscurus* | 10.22345 | 39.39972 |
| 203 | central | *T. g. obscurus* | 10.21279 | 39.09042 |
| 204 | central | *T. g. obscurus* | 10.19060 | 39.00205 |
| 205 | central | *T. g. obscurus* | 10.09447 | 39.49560 |
| 206 | central | *T. g. obscurus* | 10.08333 | 38.28333 |
| 207 | central | *T. g. obscurus* | 10.06673 | 38.21131 |
| 208 | central | *T. g. obscurus* | 10.06667 | 38.28333 |
| 209 | central | *T. g. obscurus* | 10.06617 | 39.02085 |
| 210 | central | *T. g. obscurus* | 10.06308 | 39.60314 |
| 211 | central | *T. g. obscurus* | 9.93466 | 39.23103 |
| 212 | central | *T. g. obscurus* | 9.92401 | 39.12938 |
| 213 | central | *T. g. obscurus* | 9.92128 | 38.92689 |
| 214 | central | *T. g. obscurus* | 9.91667 | 39.78333 |
| 215 | central | *T. g. obscurus* | 9.90000 | 39.78333 |
| 216 | central | *T. g. obscurus* | 9.84291 | 38.90653 |
| 217 | central | *T. g. obscurus* | 9.83989 | 38.89557 |
| 218 | central | *T. g. obscurus* | 9.83854 | 39.74226 |
| 219 | central | *T. g. obscurus* | 9.83333 | 39.78333 |
| 220 | central | *T. g. obscurus* | 9.82280 | 38.88168 |
| 221 | central | *T. g. obscurus* | 9.82199 | 39.70896 |
| 222 | central | *T. g. obscurus* | 9.82071 | 39.73438 |
| 223 | central | *T. g. obscurus* | 9.81673 | 38.89802 |
| 224 | central | *T. g. obscurus* | 9.81168 | 38.73698 |
| 225 | central | *T. g. obscurus* | 9.80000 | 38.75000 |
| 226 | central | *T. g. obscurus* | 9.79116 | 39.68223 |
| 227 | central | *T. g. obscurus* | 9.78930 | 38.99210 |
| 228 | central | *T. g. obscurus* | 9.78436 | 38.95766 |
| 229 | central | *T. g. obscurus* | 9.77978 | 39.75060 |
| 230 | central | *T. g. obscurus* | 9.75675 | 38.85727 |
| 231 | central | *T. g. obscurus* | 9.75000 | 39.75000 |
| 232 | central | *T. g. obscurus* | 9.74411 | 38.86093 |
| 233 | central | *T. g. obscurus* | 9.73816 | 39.73623 |
| 234 | central | *T. g. obscurus* | 9.73742 | 38.81266 |
| 235 | central | *T. g. obscurus* | 9.73190 | 39.74961 |
| 236 | central | *T. g. obscurus* | 9.72799 | 38.82169 |
| 237 | central | *T. g. obscurus* | 9.71682 | 38.83738 |
| 238 | central | *T. g. obscurus* | 9.71667 | 38.86667 |
| 239 | central | *T. g. obscurus* | 9.71519 | 38.84727 |
| 240 | central | *T. g. obscurus* | 9.70330 | 38.88094 |
| 241 | central | *T. g. obscurus* | 9.70000 | 39.50000 |
| 242 | central | *T. g. obscurus* | 9.70000 | 38.81667 |
| 243 | central | *T. g. obscurus* | 9.67276 | 39.50965 |
| 244 | central | *T. g. obscurus* | 9.66667 | 39.53333 |
| 245 | central | *T. g. obscurus* | 9.66667 | 39.05000 |
| 246 | central | *T. g. obscurus* | 9.66667 | 39.03333 |
| 247 | central | *T. g. obscurus* | 9.65000 | 39.75000 |
| 248 | central | *T. g. obscurus* | 9.63333 | 39.31667 |
| 249 | central | *T. g. obscurus* | 9.62176 | 39.73863 |
| 250 | central | *T. g. obscurus* | 9.58333 | 39.75000 |
| 251 | central | *T. g. obscurus* | 9.57642 | 38.91767 |
| 252 | central | *T. g. obscurus* | 9.51667 | 38.21667 |
| 253 | central | *T. g. obscurus* | 9.50000 | 38.16667 |
| 254 | central | *T. g. obscurus* | 9.49340 | 38.43410 |
| 255 | central | *T. g. obscurus* | 9.48000 | 38.43000 |
| 256 | central | *T. g. obscurus* | 9.46667 | 38.75000 |
| 257 | central | *T. g. obscurus* | 9.45260 | 39.43200 |
| 258 | central | *T. g. obscurus* | 9.44060 | 38.48510 |
| 259 | central | *T. g. obscurus* | 9.43496 | 39.53940 |
| 260 | central | *T. g. obscurus* | 9.43473 | 38.65323 |
| 261 | central | *T. g. obscurus* | 9.43010 | 38.50060 |
| 262 | central | *T. g. obscurus* | 9.41667 | 38.75000 |
| 263 | central | *T. g. obscurus* | 9.30917 | 38.73402 |
| 264 | central | *T. g. obscurus* | 9.30000 | 38.61667 |
| 265 | central | *T. g. obscurus* | 9.25000 | 38.55000 |
| 266 | central | *T. g. obscurus* | 9.16667 | 39.41667 |
| 267 | central | *T. g. obscurus* | 9.13333 | 39.10000 |
| 268 | central | *T. g. obscurus* | 9.13333 | 39.03333 |
| 269 | central | *T. g. obscurus* | 9.11667 | 39.11667 |
| 270 | central | *T. g. obscurus* | 9.08333 | 38.75000 |
| 271 | central | *T. g. obscurus* | 8.91667 | 38.61667 |
| 272 | central | *T. g. obscurus* | 8.85412 | 38.84637 |
| 273 | south | *T. g.* ssp. nov | 7.90306 | 39.27167 |
| 274 | south | *T. g.* ssp. nov | 7.74965 | 39.81547 |
| 275 | south | *T. g.* ssp. nov | 7.73912 | 39.83617 |
| 276 | south | *T. g.* ssp. nov | 7.70422 | 39.81851 |
| 277 | south | *T. g.* ssp. nov | 7.69513 | 39.81240 |
| 278 | south | *T. g.* ssp. nov | 7.69358 | 39.82556 |
| 279 | south | *T. g.* ssp. nov | 7.68000 | 40.18300 |
| 280 | south | *T. g.* ssp. nov | 7.53947 | 39.94557 |
| 281 | south | *T. g.* ssp. nov | 7.52680 | 40.02117 |
| 282 | south | *T. g.* ssp. nov | 7.51617 | 39.96753 |
| 283 | south | *T. g.* ssp. nov | 7.50983 | 39.99065 |
| 284 | south | *T. g.* ssp. nov | 7.50330 | 39.51300 |
| 285 | south | *T. g.* ssp. nov | 7.50055 | 39.99195 |


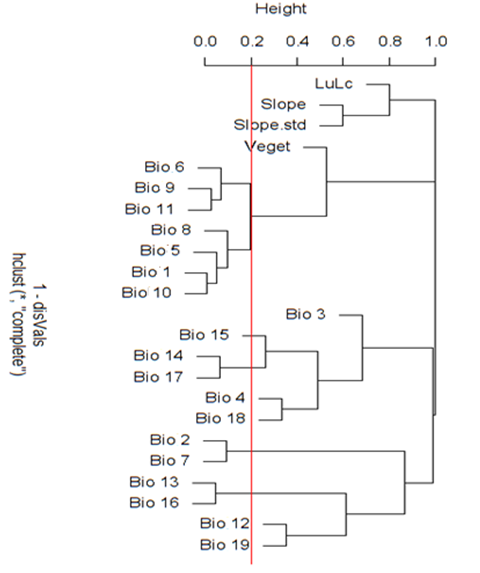


**FIGURE S1** Pairwise Pearson correlation of the predictor variables at locations of training and evaluation datasets. Redline shows a correlation coefficient r = |0.8|. Annual Mean Temperature (Bio1), Mean Diurnal Range (mean of monthly max temp - min temp; Bio2), Isothermality (Bio3), Temperature Seasonality (Bio4), Maximum Temperature of Warmest Month (Bio5), Minimum Temperature of Coldest Month (Bio6), Temperature Annual Range (Bio7), Mean Temperature of Wettest Quarter (Bio8), Annual Precipitation (Bio12), Precipitation of Wettest Month (Bio13), Precipitation of Driest Month (Bio14), Precipitation Seasonality (Coefficient of Variation) (Bio15), Precipitation of Wettest Quarter (Bio16), Precipitation of Driest Quarter (Bio17), Precipitation of Warmest Quarter (Bio18), Precipitation of Coldest Quarter (Bio19), Slope, Slope Standard Deviation (Slope. Std), Land use land cover change (LuLc) and Vegetation.


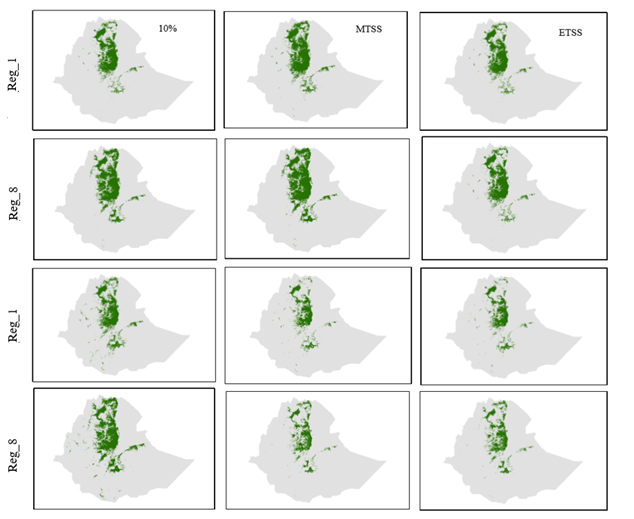


**Figure S2** Distribution and extent of suitable habitat of T. gelada produced using two levels of model complexity (regularization multiplier value = 1 (Reg_1) and 8 (Reg_8)) and three cut-off threshold values: 10% (10 percent omission rate), MTSS (maximum test sensitivity and specificity), and ETSS (equal test sensitivity and specificity). The maps in the upper two rows were produced by generating pseudoabsence points within the elevation ranges of the occurrence points of T. gelada (2018 – 4219 m asl), while the lower two rows were produced by generating pseudoabsence points using a bias file.


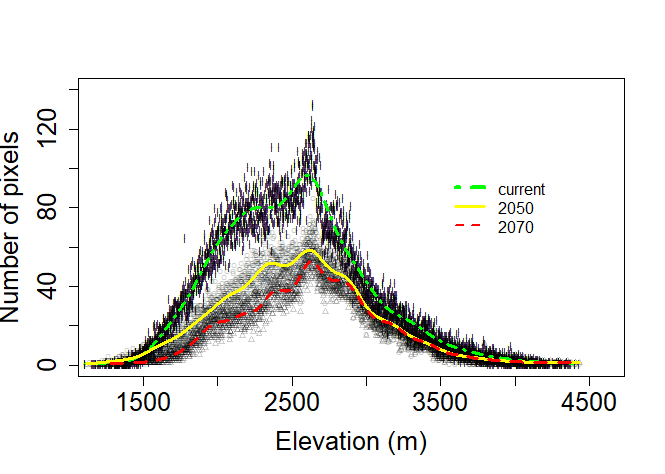


**FIGURE S3** Predicted size of suitable habitats of gelada across an elevation gradient under current and future climates (2050 and 2070). The x-axis represents altitude (m) and the y-axis represents 1 km × 1 km grid cell counts.


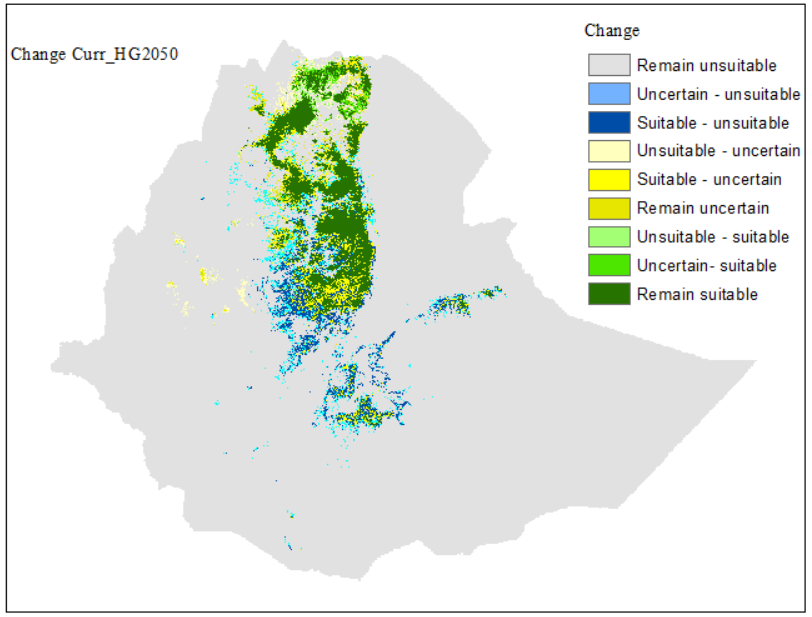


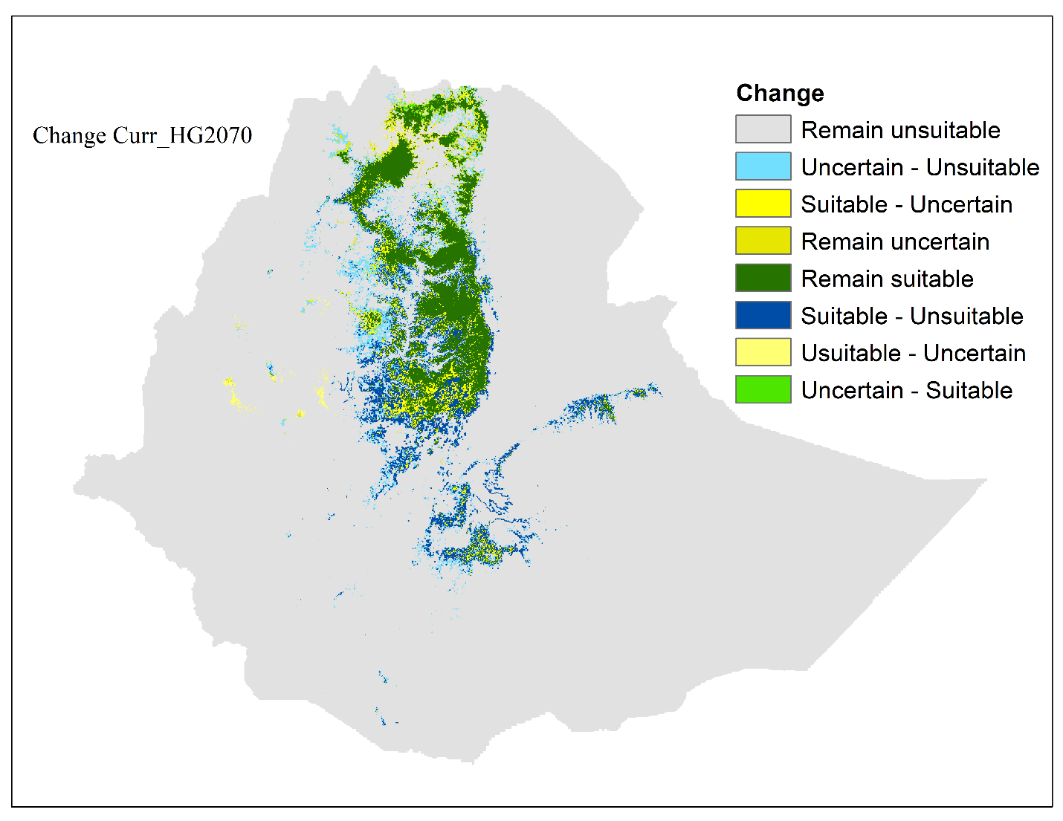


**FIGURE S4** Changes in distribution and extent of suitable gelada habitat. The habitat suitability maps show predicted losses and gains by comparing current and future projections (curr_2050 and curr_2070).
